# Supplementary material for: Dynamic interactions between the RNA chaperone Hfq, small regulatory RNAs, and mRNAs in live bacterial cells
Source: eLife. 2021 Feb 22;10:e64207. doi: 10.7554/eLife.64207 (PMC7987339; doi:10.7554/eLife.64207)
Supplement: Source code 1. [file elife-64207-code1.zip › sptPALM_MSD_osd2/Tutorial.docx]

Tutorial of how to use the tracking analysis and “MSD”/”*osd* square” calculation package

(Feb, 2021), Seongjin Park (contact: [prelist@gmail.com](mailto:prelist@gmail.com))

This package was originally written by John C. Crocker (Univ. of Pennsylvania) and was modified by Yong Wang (Univ. of Arkansas). Seongjin Park did not change the algorithm, but added several interface codes.

The source codes have been running in MatLab R2019b version, but should be compatible with most of earlier or later versions too.

The source codes are for the dataset of 5.76 ms time interval between two consecutive frames, and with the trajectory forming criteria of 400 nm and 5 padded consecutive frames (or 400 nm and 3 consecutive frames without padding). For one’s specific experiment with different parameters, please note “Additional Notes” at the end of this tutorial.

------------How to calculate ensemble mean squared displacement (MSD) from multiple datasets -----

Unzip “sptPALM_Wang” package and place the whole folder somewhere MatLab can easily access.

1. Include the directory of this folder (not with sub-folders) in Matlab’s Path (Home🡪 Set Path🡪Add Folder).
2. Each output file (.txt file) from sptPALM analysis should look like a matrix of

[x,y,z,color_code,frame_number]

, where (x,y,z) is the coordinate of each spot (unit: nm), and “frame_number” is the imaging frame number where a certain spot was detected. Make the color_code any integer. The test data sets are in the sub-folder, “Test_data.” (Assume that there are three data sets from sptPALM.)

Test_data_sptPALM_x_y_z_color_frame_0.txt

Test_data_sptPALM_x_y_z_color_frame_1.txt

Test_data_sptPALM_x_y_z_color_frame_2.txt

1. From “ensemble_MSD_cal_for_each_dataset” folder, copy T0, T1 and T2 folders and paste them inside of “Test_data” folder.
2. Place “Test_data_sptPALM_x_y_z_color_frame_0.txt” in Tr0 folder, “Test_data_sptPALM_x_y_z_color_frame_1.txt” in Tr1 folder, and “Test_data_sptPALM_x_y_z_color_frame_2.txt” in Tr2 folder.
3. Open MatLab, and set the working directory to be Tr0. Type

“script(400,5)”

in the Command Window and hit the Enter key. (This is for detecting/forming trajectories with 400 nm distance cutoff and with 5 consecutive frames.)

1. A pop-up window will appear. Select “Test_data_sptPALM_x_y_z_color_frame_0.txt” and click “Open.”
2. Once done, type

“built_msds_2D”

in the Command Window and hit the Enter key. “mean_MSD.dat” file is generated as an outcome, and this file contains MSD calculated for the single dataset, as a format of [time, MSD, standard error].

1. Repeat 6~8 in Tr1 and Tr2 folders.
2. In the parent directory of the sptPALM datasets, crease a folder named “InterP_result_comb_400_5”. Copy “result_tracking_400_min_5.txt” from Tr0 folder and paste it here. Rename the file as “result_tracking_0.txt”. Do the same thing from Tr1 and Tr2 folders, so that in “InterP_result_comb_400_5” folder, there are three files as

result_tracking_0.txt

result_tracking_1.txt

result_tracking_2.txt

(Note: “result_tracking_400_min_5.txt” contains tracking data with padding.)

1. From “ensemble_MSD_after_comb” folder, copy the following files

combining_result_tracking_2D_2.m

built_msds_2D.m

create_finalTraj_2D.m

MSD_ave_3D

MSD_fit_3D

Onestep_MSD_2D

sptPALM_calc_msds

and paste them in “InterP_result_comb_400_5” folder.

1. Change the working directory of MatLab to “InterP_result_comb_400_5” folder, and type

“combining_result_tracking_2D_2”

In the Command Window and hit the Enter key.

“result_comb.txt” file is generated as an outcome. Rename it as “InterP_result_comb_400_5.txt”.

1. Type

“built_msds_2D”

in the Command Window and hit the Enter key. “mean_MSD.dat” file is generated as an outcome, and this file contains MSD (unit: µm^2^/s) calculated from the combined dataset, as a format of [time, MSD, standard error]. Further fitting can be done this outcome to extract the ensemble diffusion coefficient, either by MatLab or a different software such as Origin Pro.

---------------------How to calculate one-step displacement square (*osd*^2^) from multiple datasets (assuming that all the above steps were done earlier) ------------------------

1. Open MatLab, and set the working directory to be Tr0. Type

“script(400,3)”

in the Command Window and hit the Enter key. (This is for detecting/forming trajectories with 400 nm distance cutoff and with 3 consecutive frames.)

1. A pop-up window will appear. Select “Test_data_sptPALM_x_y_z_color_frame_0.txt” and click “Open.”
2. Repeat 1~2 in Tr1 and Tr2 folders.
3. In the parent directory of the sptPALM datasets, crease a folder named “noInterP_result_comb_400_3”. Copy “noInterP_result_tracking_400_min_3.txt” from Tr0 folder and paste it here. Rename the file as “result_tracking_0.txt”. Do the same thing from Tr1 and Tr2 folders, so that in “noInterP_result_comb_400_3” folder, there are three files as

result_tracking_0.txt

result_tracking_1.txt

result_tracking_2.txt

(Note: “noInterP_result_tracking_400_min_3.txt” contains tracking data without padding.)

1. From “ensemble_MSD_after_comb” folder, copy the following files

combining_result_tracking_2D_2.m

built_msds_2D.m

create_finalTraj_2D.m

MSD_ave_3D

MSD_fit_3D

Onestep_MSD_2D

sptPALM_calc_msds

and paste them in “noInterP_result_comb_400_3” folder.

1. Change the working directory of MatLab to “noInterP_result_comb_400_3” folder, and type

“combining_result_tracking_2D_2”

in the Command Window and hit the Enter key.

“result_comb.txt” file is generated as an outcome. Rename it as “noInterP_result_comb_400_3.txt”.

1. Type

“osd_2D_comb”

in the Command Window and hit the Enter key.

“onestep_MSD_list_min_3.dat” is generated. The fourth column of this output file has *osd*^2^ (unit: µm^2^) values from individual trajectories. Fitting software such as Origin Pro can be used to fit the cumulative distribution of *osd*^2^ to extract sub populations of states.

-------------------------- Additional Notes---------------------------------------------------------------------

In the case of applying this package to the dataset with different time intervals and different criteria for forming trajectories, conduct the following steps.

For changing time interval values,

1. Open “build_msds_2D.m” codes from “ensemble_MSD_after_comb” folder and all Tr0..Tr14 folders in “ensemble_MSD_cal_for_each_dataset” folder, and change 0.00576 to the correct time interval.
2. Open “MSD_ave_3D.m” codes the same folders, and change

time = [0.00576;0.01152;0.01728;0.02304;0.0288;0.03456; 0.04032;0.04608;0.05184];

to the correct time intervals.

For changing distance cut-off criteria,

1. Open “build_msds_2D.m” codes from “ensemble_MSD_after_comb” folder and all Tr0..Tr14 folders in “ensemble_MSD_cal_for_each_dataset” folder, and change 400 to the correct distance cut-off value (unit: nm).
2. Open “osd_2D_comb.m” from “ensemble_MSD_after_comb” folder and “osd_2D_individual_file.m” from all Tr0..Tr14 folders in “ensemble_MSD_cal_for_each_dataset” folder, and change 400 to the correct distance cut-off value (unit: nm).

For changing different number of consecutive frames other than 5 or 3, open the same files as above for distance cut-off, but change either 5 or 3 to the correct number.
